# Association between hearing loss, tinnitus, and atherosclerotic cardiovascular disease among American adults: A cross-sectional study

**DOI:** 10.1097/MD.0000000000050087

**Published:** 2026-08-07

**Authors:** Luniao Ma, Xuhui Huang, Yangyang Chen, Huimin Zhang, Yao Zeng, Xushan Chen, Rucheng Huang, Xiaona Tang

**Affiliations:** a Nursing Department, The Seventh Clinical College of Guangzhou University of Chinese Medicine, Shenzhen, China.

**Keywords:** ASCVD, hearing loss, NHANES, tinnitus

## Abstract

The interconnections between hearing loss, tinnitus, and atherosclerotic cardiovascular disease (ASCVD) remain incompletely elucidated. This study sought to investigate the associations of ASCVD prevalence with hearing loss and tinnitus. We analyzed data from the National Health and Nutrition Examination Survey (2015–2018), encompassing 19,225 US adults aged 20 years and older in this cross-sectional study. Multivariable logistic regression models were employed to examine potential links between ASCVD and both hearing loss and tinnitus. Moreover, we performed subgroup analyses and interaction tests to explore if the relationship was stable in different subgroups. Among 4320 adults included in this study (mean age, 49.8 ± 17.5 years), 452 (10.5%) had ASCVD and 3868 (89.5%) did not have ASCVD. Compared with participants without hearing loss, those with hearing loss had a higher prevalence of ASCVD (unadjusted odds ratio [OR] = 3.62, 95% confidence interval [CI] = 2.96–4.42, *P* < .001; adjusted OR = 1.49, 95% CI = 1.18–1.88, *P* < .001). Similarly, tinnitus was independently associated with higher odds of ASCVD (unadjusted OR = 2.40, 95% CI = 1.92–2.99, *P* < .001; adjusted OR = 1.63, 95% CI = 1.27–2.09, *P* < .001). This study of US adults revealed robust associations between hearing loss, tinnitus, and ASCVD prevalence.

## 1. Introduction

Atherosclerotic cardiovascular disease (ASCVD) encompasses coronary, cerebrovascular, and peripheral arterial conditions, and it remains the leading global cause of mortality, responsible for around 17.9 million deaths annually.^[1,2]^ The persistent rise in mortality rates establishes ASCVD as a critical public health challenge worldwide. This condition imposes substantial personal health impairment, reduced life expectancy, and heightened socioeconomic burdens. Emerging evidence suggests that occupational noise exposure may be a potential risk factor for ASCVD,^[3,4]^ particularly in male and elderly demographic groups. Moreover, chronic noise exposure is associated with an increased risk of hearing impairment.

Hearing loss and tinnitus constitute primary manifestations of auditory impairment with global prevalence. Their etiology is multifactorial, encompassing chronic noise exposure, suboptimal management of chronic conditions, and population aging. Hearing loss is categorized into 3 principal types: conductive, sensorineural, and mixed hearing loss.^[5]^ Among these, sensorineural hearing loss (SNHL) and subjective tinnitus represent growing public health concerns globally. Current estimates indicate approximately 1.5 billion individuals are affected by these conditions, with an additional 740 million experiencing substantial quality-of-life impairment. Furthermore, SNHL prevalence demonstrates a significant age-dependent increase,^[6,7]^ highlighting its clinical significance in aging populations.

Although hearing impairment and cardiovascular disease (CVD) represent distinct clinical entities, emerging evidence suggests that otoacoustic mapping may serve as a predictor for cardiovascular health status. Epidemiological studies demonstrate associations between cardiovascular risk factors and hearing loss in male populations.^[8,9]^ The latest epidemiological studies reveal a potential pathophysiological link between hearing impairment and CVDs. Vascular disorders (such as atherosclerosis) and systemic inflammation may directly cause cochlear damage and hearing dysfunction.^[10,11]^ Recent clinical evidence has documented ischemic events following cochlear implantation in patients without established cardiovascular risk factors, underscoring the necessity for this study.^[12]^

Several studies have reported associations between hearing impairment and CVDs; current evidence predominantly derives from limited observational investigations, isolated case reports, and Asian populations. For instance, a 2023 investigation identified carotid atherosclerotic lesions in patients with SNHL, corroborating the vascular hypothesis.^[13]^ However, a comprehensive cross-sectional analysis evaluating these interrelationships is currently lacking. To resolve this evidence deficit, we undertook an extensive population-level cross-sectional analysis exploring associations between chronic cardiovascular disorders and auditory impairments specifically: hearing loss and tinnitus. In addition, we performed severity-stratified analyses of ASCVD-related hearing impairment and subgroup assessments to identify population-specific correlations.

National Health and Nutrition Examination Survey (NHANES) uses a cross-sectional, population-based design to gather health and nutritional data from households across the United States. Its complex multistage probability sampling design ensures that the estimates are nationally representative. However, the NHANES database has not been systematically utilized to explore the associations among hearing loss, tinnitus, and ASCVD. Thus, our study aims to examine the relationships among ASCVD, hearing loss, and tinnitus. Clarifying these associations may provide epidemiological evidence for identifying populations with overlapping auditory and cardiovascular health concerns and may inform future risk-stratified prevention strategies.

## 2. Materials and methods

### 2.1. Study design and participants

Conducted by the National Center for Health Statistics, this represents a large-scale cross-sectional initiative designed to evaluate health and nutritional parameters among noninstitutionalized US adults and children. Comprehensive demographic, socioeconomic, laboratory, dietary, and physiological data were systematically collected. Trained personnel administered interviews, questionnaires, and conducted physical examinations within mobile examination centers, with concomitant collection of biological specimens for laboratory analyses. Ethical approval for the NHANES protocol was granted by the National Center for Health Statistics Institutional Review Board, and written informed consent was secured from all participants. Secondary analyses were exempted from further institutional review under federal provisions. We can access the NHANES data via the NHANES website.

This cross-sectional study employed NHANES data from the 2015 to 2018 cycles. These data enabled evaluation of interconnections among ASCVD, auditory deficits (hearing loss and tinnitus), and cardiovascular risk. Our analysis specifically targeted interview respondents aged 20 years and older. This age threshold was established to align with the NHANES adult survey framework, as comprehensive medical history and audiological questionnaires are consistently administered to the population starting at this age. Furthermore, while clinical ASCVD events are less prevalent in early adulthood, histological evidence confirms that the initiation of subclinical atherosclerosis often occurs as early as the second decade of life.^[14]^ Including participants from age 20 onwards thus ensures a representative sample of the US adult population and allows for a more complete assessment of cardiovascular and auditory associations across the full adult life course. Following the exclusion of records with incomplete primary variables, 8994 participants remained. Subsequent exclusion of pregnant individuals and cases with incomplete covariate data established the final participants (n = 4320).

### 2.2. The diagnosis of ASCVD

ASCVD encompasses patients presenting with acute coronary syndrome, myocardial infarction, stable/unstable angina, coronary or peripheral arterial revascularization, stroke, transient ischemic attack, peripheral arterial disease, and other atherosclerosis-related pathologies.^[15]^ We ascertained whether participants had received an ASCVD diagnosis by evaluating self-reported responses to this medical history questionnaire: “Has a doctor or other health professional informed you of angina, heart attack, stroke, or coronary heart disease?” To ensure clinical accuracy and distinguish established disease from subclinical symptoms, NHANES protocols strictly employ a “doctor-diagnosed” criterion. According to the interviewer manual, self-diagnosed cases or reports from non-healthcare professionals are systematically excluded, ensuring that the data reflect confirmed clinical events rather than subjective health complaints.

### 2.3. Definitions of hearing loss and tinnitus

Study inclusion required participant-reported hearing status using validated NHANES instruments,^[16]^ with severity categorized by numerical scale: 3 (mild hearing loss), 4 (moderate), 5 (severe), or 6 (deafness). The tinnitus case definition derived from affirmative responses to the protocol-specified item: “Have you experienced tinnitus, ear ringing, or buzzing sounds lasting ≥5 minutes in the preceding 12 months?”^[17]^ To maintain data reliability among participants with hearing loss, NHANES implements specific communication safeguards. During the interview, trained staff utilize printed hand cards that display each question and its corresponding response categories in a clear, large-font format. This dual-sensory (visual and auditory) approach, conducted in a private and quiet setting within the mobile examination center, minimizes the risk of misinterpretation or communication barriers, ensuring the integrity of the self-reported information.

### 2.4. The covariates

Within the NHANES framework, potential covariates were assessed: sex, age, race/ethnicity, education level, marital status, poverty-income ratio (PIR), body mass index (BMI), hypertension, smoking, and depression. Race/ethnicity was stratified into: Mexican American, other Hispanic, non-Hispanic White, non-Hispanic Black, and other race. Educational level was categorized as: <9 years, 9 to 12 years, and >12 years. Marital status was divided into married or living with partner: single, divorced, or widowed. PIR was stratified into 3 categories: low (PIR < 1.30), medium (PIR > 1.3–3.5), and high (PIR > 3.50). BMI was derived from weight (kg)/height (m)^2^, stratified as: <25.0 (normal) and ≥25.0 kg/m^2^ (overweight and obese).^[18]^ Smoking status was classified based on self-reported lifetime cigarette exposure (≥100 cigarettes) and current smoking status.^[19]^ Depression severity was measured using the validated 9-item patient health questionnaire instrument, with scores ≥10 establishing clinically relevant depressive symptomatology.^[20]^ Comprehensive documentation for all variables is publicly accessible via the NHANES online catalogue: https://wwwn.cdc.gov/Nchs/Nhanes/continuousnhanes.

### 2.5. Statistical analysis

Continuous variables were presented as mean ± standard deviation for normally distributed data or as median (interquartile range) for non-normally distributed data. Intergroup comparisons were conducted using one-way analysis of variance for normally distributed data, Kruskal–Wallis test for skewed data, and chi-square test for categorical variables. Table 1 summarizes the baseline characteristics of participants with ASCVD compared to those without ASCVD.

**Table 1:** Baseline characteristics of the participants according to atherosclerotic cardiovascular disease history.

ASCVD	Total (n = 4320)	History (n = 452)	No history (n = 3868)	*P*
Sex, n (%)				<.001
Male	2154 (49.9)	269 (59.5)	1885 (48.7)	
Female	2166 (50.1)	183 (40.5)	1983 (51.3)	
Age, mean ± SD	49.8 ± 17.5	66.2 ± 12.5	47.9 ± 17.0	<.001
Race/ethnicity, n (%)				<.001
Mexican American	692 (16.0)	34 (7.5)	658 (17)	
Other Hispanic	517 (12.0)	45 (10)	472 (12.2)	
Non-Hispanic White	1580 (36.6)	230 (50.9)	1350 (34.9)	
Non-Hispanic Black	905 (20.9)	107 (23.7)	798 (20.6)	
Other race	626 (14.5)	36 (8)	590 (15.3)	
Education level, n (%)				.057
<9	905 (20.9)	108 (23.9)	797 (20.6)	
9–12	990 (22.9)	114 (25.2)	876 (22.6)	
>12	2425 (56.1)	230 (50.9)	2195 (56.7)	
Married status, n (%)				.006
Married or living with partner	2646 (61.3)	250 (55.3)	2396 (61.9)	
Single, divorced, or widowed	1674 (38.8)	202 (44.7)	1472 (38.1)	
PIR, n (%)				.037
≤1.3	1298 (30.0)	155 (34.3)	1143 (29.6)	
>1.3	3022 (70.0)	297 (65.7)	2725 (70.4)	
BMI, mean ± SD	29.7 ± 7.1	31.0 ± 7.3	29.5 ± 7.1	<.001
Hypertension, n (%)				<.001
Yes	1608 (37.2)	331 (73.2)	1277 (33)	
No	2712 (62.8)	121 (26.8)	2591 (67)	
Depression, n (%)				<.001
Yes	430 (10.0)	78 (17.3)	352 (9.1)	
No	3890 (90.0)	374 (82.7)	3516 (90.9)	
Smoking, n (%)				<.001
Yes	1882 (43.6)	287 (63.5)	1595 (41.2)	
No	2438 (56.4)	165 (36.5)	2273 (58.8)	
Hearing loss, n (%)				<.001
Yes	1016 (23.5)	219 (48.5)	797 (20.6)	
No	3304 (76.5)	233 (51.5)	3071 (79.4)	
Tinnitus, n (%)				<.001
Yes	712 (16.5)	134 (29.6)	578 (14.9)	
No	3608 (83.5)	318 (70.4)	3290 (85.1)	

ASCVD = atherosclerotic cardiovascular disease, BMI = body mass index, PIR = poverty-income ratio, SD = standard deviation.

Unweighted multivariable logistic regression models were applied to evaluate the associations between hearing loss, tinnitus, and the prevalence of ASCVD. We did not apply sampling weights because the primary objective of this study was to evaluate independent associations rather than to estimate national population prevalence. Instead, to account for the complex survey design, key demographic variables (e.g., age, sex, and race/ethnicity) were included as adjusting covariates in the regression models. Results were expressed as odds ratios (ORs) with 95% confidence intervals (CIs). Four models were constructed. Model 1 was adjusted for age, while Model 2 included adjustments for age, sex, and race/ethnicity. Model 3 was adjusted for all factors in Model 2, with the addition of PIR, education level, and marital status. Model 4 further included BMI, depression, smoking, and hypertension. Subgroup analyses were performed using the fully adjusted model, and *P* values for interaction were obtained by testing the interaction terms in the fully adjusted models. Additive interaction between hearing loss and tinnitus was evaluated using the relative excess risk due to interaction (RERI), attributable proportion due to interaction (AP), and synergy index (SI), with statistical significance determined from their 95% CIs. Multiplicative interaction was assessed by including a hearing loss × tinnitus interaction term in the fully adjusted model.

All statistical procedures were executed in R version 4.5.1 (R Foundation for Statistical Computing, Vienna, Austria) and Free Statistics software version 2.3 (Beijing Free Clinical Medical Technology Co., Ltd., Beijing, China) in this study. All statistical tests were conducted using a two-sided approach. *P* < .05 was considered statistically significant.

## 3. Results

### 3.1. Study population

**Figure 1.**
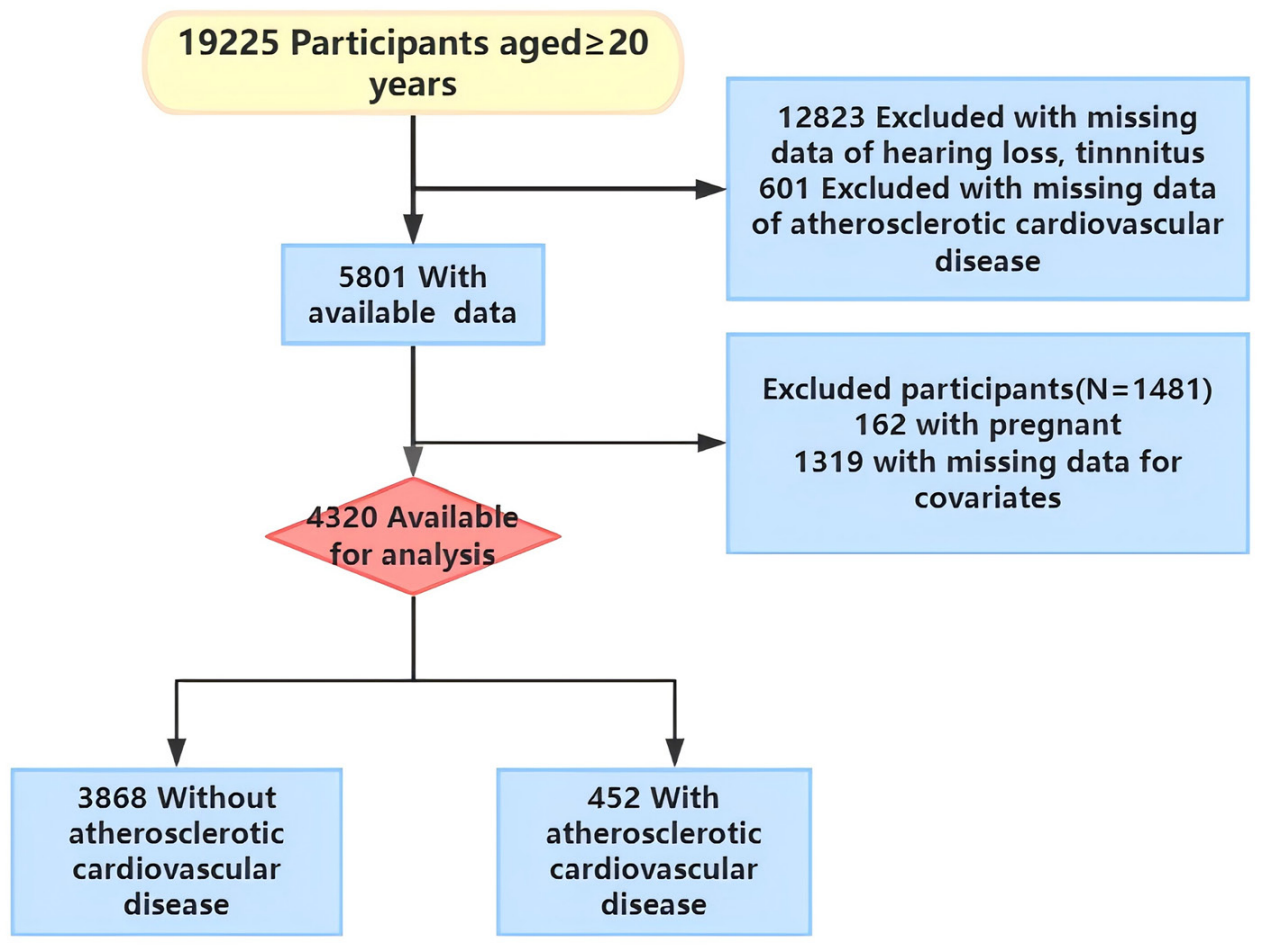
Flowchart of the selection of study participants.

A total of 19,225 participants aged 20 years or older were initially recruited and completed interviews. Participants were excluded due to missing data on hearing loss or tinnitus (n = 12,823), absence of ASCVD information (including coronary heart disease, angina, heart attack, and stroke; n = 601), pregnancy (n = 162), or incomplete covariate data (n = 1319). Following the implementation of exclusionary criteria, the final analytical cohort comprised 4320 qualifying individuals from NHANES 2015 to 2018. The participant selection process is shown in Figure 1.

### 3.2. Baseline characteristics

Table 1 summarizes the baseline characteristics of the 4320 study participants categorized by ASCVD history. Significant demographic and clinical differences were identified between the 2 groups. Participants with a history of ASCVD (n = 452) were significantly older (66.2 ± 12.5 years, *P* < .001) and had a higher likelihood of being male (59.5%, *P* < .001) than those without ASCVD (n = 3868). The racial distribution differed significantly (*P* < .001), with a higher proportion of non-Hispanic White individuals in the ASCVD group (50.9%). The ASCVD group displayed higher rates of hypertension (73.2%, *P* < .001), depression (17.3%, *P* < .001), and current smoking (63.5%, *P* < .001). Socioeconomic factors demonstrated modest associations; the ASCVD group had lower PIR and a lower proportion of individuals who were single, divorced, or widowed. The education level approached but did not achieve statistical significance (*P* = .057).

### 3.3. Association between hearing loss, tinnitus, and ASCVD

Table 2 displays the multivariable-adjusted associations of hearing loss and tinnitus with prevalent ASCVD. Multivariable logistic regression revealed significant associations between hearing loss and ASCVD in this study. Participants with hearing loss showed a higher prevalence of ASCVD compared to those without hearing loss (Table 2). In Model 1, we adjusted for age; Model 2 adjusted for age, sex, and race/ethnicity; Model 3 was further adjusted for education level, marital status, and PIR in addition to the variables included in Model 2; and after adjusting for all covariates in Model 4 (age, sex, race/ethnicity, education level, marital status, PIR, BMI, smoking status, depression, and hypertension), hearing loss remained significantly associated with higher odds of ASCVD (OR = 1.49, 95% CI = 1.18–1.88, *P* < .001). Similarly, tinnitus was also independently associated with higher odds of ASCVD after adjustment for the same covariates (OR = 1.63, 95% CI = 1.27–2.09, *P* < .001; Table 2).

**Table 2:** Multivariable-adjusted associations of hearing loss and tinnitus with prevalent ASCVD.

	Model 1		Model 2		Model 3		Model 4	
	OR (95% CI)	*P*	OR (95% CI)	*P*	OR (95% CI)	*P*	OR (95% CI)	*P*
ASCVD								
Hearing loss−	Reference		Reference		Reference		Reference	
Hearing loss+	1.79 (1.44–2.23)	<.001	1.76 (1.40–2.21)	<.001	1.74 (1.38–2.18)	<.001	1.49 (1.18–1.88)	<.001
ASCVD								
Tinnitus−	Reference		Reference		Reference		Reference	
Tinnitus+	1.89 (1.5–2.4)	<.001	1.89 (1.48–2.4)	<.001	1.88 (1.48–2.39)	<.001	1.63 (1.27–2.09)	<.001

Model 1: adjusted for age. Model 2: adjusted for age, sex, and race/ethnicity. Model 3: adjusted for all the factors in Model 2 plus education level, marital status, and PIR. Model 4: adjusted for all the factors in Model 3 plus BMI, smoking, depression, and hypertension disease.

ASCVD = atherosclerotic cardiovascular disease, BMI = body mass index, CI = confidence interval, OR = odds ratio.

### 3.4. Subgroup analyses

**Figure 2.**
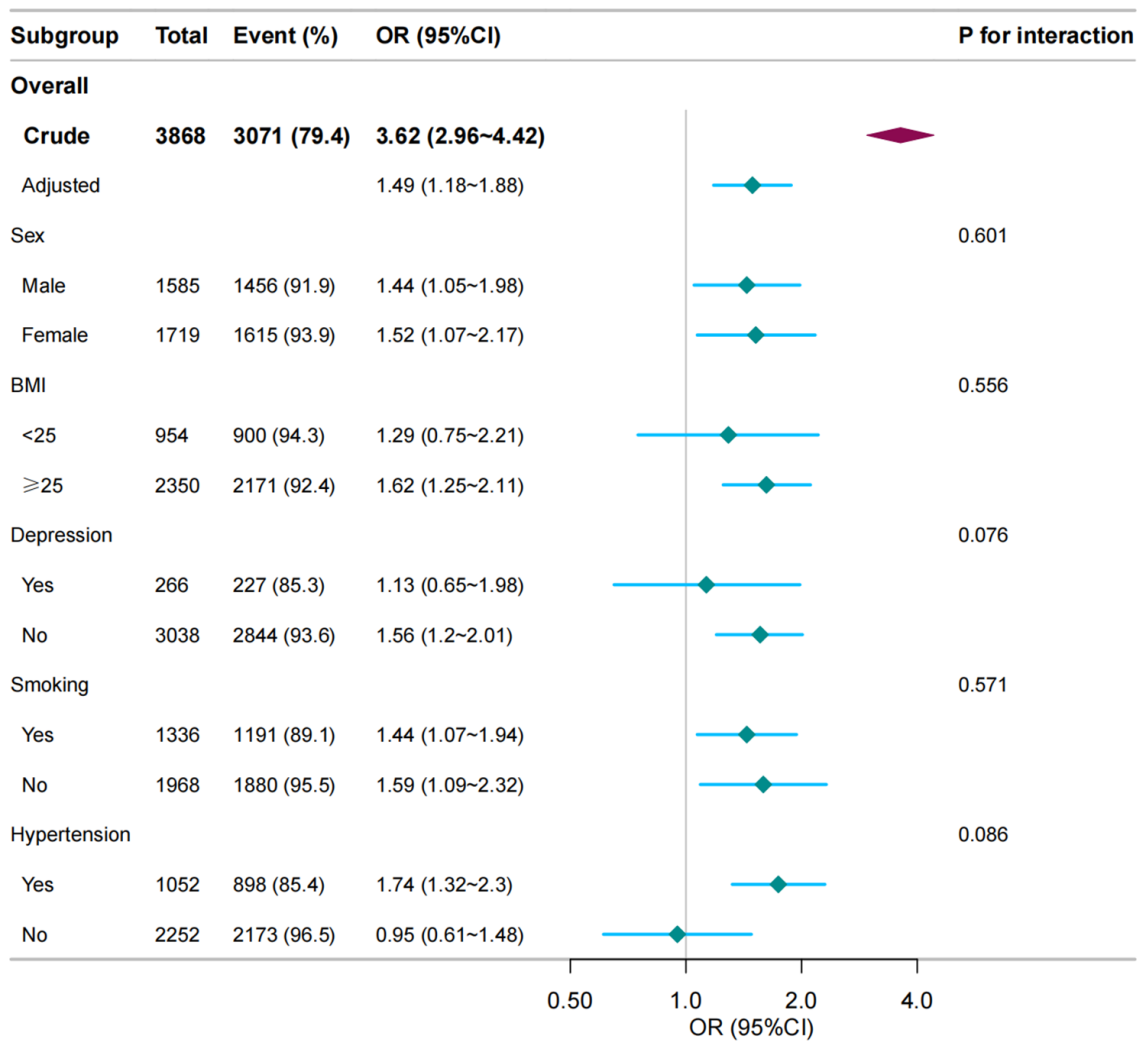
Subgroup analysis of hearing loss and atherosclerotic cardiovascular disease. BMI = body mass index, CI = confidence interval, OR = odds ratio.

**Figure 3.**
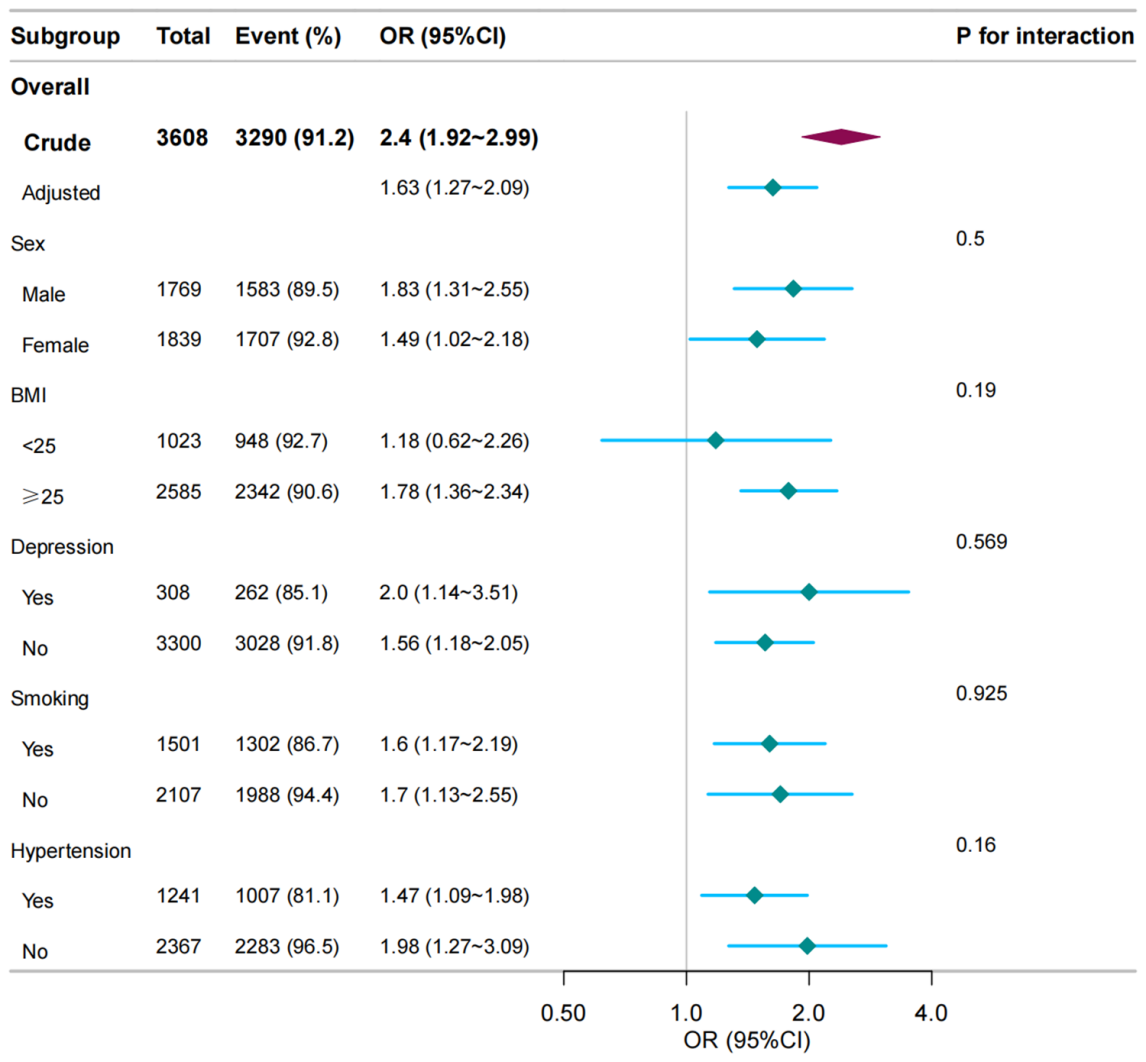
Subgroup analysis of tinnitus and atherosclerotic cardiovascular disease. BMI = body mass index, CI = confidence interval, OR = odds ratio.

In addition, subgroup analyses and effect modification analyses were conducted to evaluate whether the associations of hearing loss and tinnitus with prevalent ASCVD differed across selected subgroups, including sex, BMI, depression status, smoking status, and hypertension status (Figs. 2 and 3). For hearing loss, the overall fully adjusted OR was 1.49 (95% CI = 1.18–1.88). Sex-stratified analysis showed comparable associations in males (OR = 1.44, 95% CI = 1.05–1.98) and females (OR = 1.52, 95% CI = 1.07–2.17), with no significant interaction by sex (*P* for interaction = .601). Similarly, no statistically significant effect modification was observed by BMI, depression status, smoking status, or hypertension status, with all *P* values for interaction > .05 (Fig. 2).

For tinnitus, the overall fully adjusted OR was 1.63 (95% CI = 1.27–2.09). Subgroup analyses showed that the association between tinnitus and prevalent ASCVD was generally consistent across sex, BMI, depression status, smoking status, and hypertension status. No statistically significant effect modification was observed across these subgroups, with all *P* values for interaction > .05 (Fig. 3).

### 3.5. Additive interaction

This study investigated the joint association and interaction between hearing loss and tinnitus in relation to ASCVD. In the fully adjusted joint-exposure model, compared with participants without hearing loss or tinnitus, those with coexisting hearing loss and tinnitus had significantly higher odds of ASCVD (OR = 1.98, 95% CI = 1.43–2.73, *P* < .001).

For the additive interaction analysis, the RERI was 0.55 (95% CI = −0.03 to 1.14), and the AP was 0.28 (95% CI = −0.02 to 0.58). The 95% CIs for both RERI and AP included the null value of 0, indicating limited evidence for additive interaction. The SI was 2.31 (95% CI = 0.51–10.44), and its 95% CI included the null value of 1. No statistically significant multiplicative interaction was observed (OR = 1.36, 95% CI = 0.82–2.24, *P* = .24; Table 3).

**Table 3:** Joint association and interaction analyses of hearing loss and tinnitus in relation to prevalent ASCVD.

Analysis	OR or interaction index (95% CI)	*P*
Joint exposure analysis		
Neither hearing loss nor tinnitus	Reference	–
Tinnitus only	1.31 (0.93 to 1.86)	.12
Hearing loss only	1.11 (0.72 to 1.70)	.63
Both hearing loss and tinnitus	1.98 (1.43 to 2.73)	<.001
Additive interaction indices		
RERI	0.55 (−0.03 to 1.14)	–
AP	0.28 (−0.02 to 0.58)	–
SI	2.31 (0.51 to 10.44)	–
Multiplicative interaction		
Hearing loss × tinnitus	1.36 (0.82 to 2.24)	.24

Joint exposure and interaction analyses were based on the fully adjusted model, including age, sex, race/ethnicity, education level, marital status, PIR, BMI, smoking status, depression, and hypertension.

AP = attributable proportion due to interaction, ASCVD = atherosclerotic cardiovascular disease, BMI = body mass index, CI = confidence interval, OR = odds ratio, PIR = poverty-income ratio, RERI = relative excess risk due to interaction, SI = synergy index.

## 4. Discussion

This study investigated the association between hearing loss, tinnitus, and the prevalence of ASCVD. Subjects with hearing loss demonstrated a 49% greater prevalence of ASCVD relative to those without hearing loss. Individuals with tinnitus exhibited a 63% higher prevalence of ASCVD relative to those without tinnitus.

Previous meta-analyses have established an association between hearing loss and CVD, mediated by vascular pathways.^[21]^ A clinical study demonstrated that hearing impairment influences CVD incidence in a Chinese cohort.^[22]^ A UK prospective cohort study demonstrated a 4-fold increase in cardiovascular event risk associated with tinnitus, consistent with our findings.^[23]^ After adjusting for confounders, sensorineural bilateral high-frequency hearing loss was associated with CVD in a Canadian study.^[24]^ In a Korean cohort study, Park et al^[25]^ reported a 1.17-fold elevated risk of cardiovascular and cerebrovascular diseases among individuals with hearing impairment, which is directionally consistent with our finding that hearing loss was associated with higher odds of ASCVD. Similarly, Yang et al^[26]^ observed increased CVD risks associated with hearing loss in middle-aged and elderly Chinese cohorts, consistent with our findings on ASCVD. Unlike prior investigations, our study employed NHANES data with multivariate regression to adjust for confounders, enhancing broader applicability. Subgroup analyses confirmed the robustness of these results. Although focusing on the previously unstudied US demographic in American research, our findings reveal conflicting evidence. Notably, a Norwegian study observed no tinnitus-cardiovascular association, potentially reflecting population differences.^[27]^ Similarly, recent data from the Gutenberg Health Study reported that tinnitus is not associated with cardiovascular risk factors or mortality.^[28]^ The discrepancy among these cohorts highlights the ongoing debate regarding tinnitus and vascular health, which further illustrates the importance of our research utilizing a large, nationally representative US sample to provide more definitive evidence.

In addition to the independent associations, our joint-exposure analysis showed that participants with coexisting hearing loss and tinnitus had higher odds of ASCVD than those without either condition. However, the evidence for a statistical interaction between hearing loss and tinnitus was not definitive. Although the point estimates of RERI, AP, and SI suggested a possible positive additive interaction, the 95% CIs for RERI and AP included 0, and the 95% CI for SI included 1. In addition, no significant multiplicative interaction was observed. Therefore, the coexistence of hearing loss and tinnitus should be interpreted as identifying a subgroup with a higher ASCVD burden rather than as conclusive evidence of a synergistic effect.

Although the precise mechanism underlying hearing dysfunction in ASCVD patients remains unclear, several hypotheses exist. Atherosclerosis-related pro-inflammatory cytokines (interleukin-6, tumor necrosis factor-alpha, C-reactive protein) may exacerbate cochlear oxidative stress.^[29]^ Furthermore, updated evidence strongly emphasizes that systemic redox imbalance and intense oxidative stress are central drivers in the pathogenesis and progression of CVDs.^[30]^ Because the inner ear is highly vulnerable to systemic inflammatory markers, this global oxidative damage can easily disrupt cochlear homeostasis. The section of the cochlea responsible for transmitting low-frequency sounds has the most distant blood supply, rendering it particularly sensitive to ischemia in patients with potential CVD.^[31]^ Histopathological studies have shown that microvascular disease impacts both high-frequency and low-frequency regions of the cochlea. In a study of temporal bone cadavers, cochlear degeneration was observed at both the apical and basal turns, resulting in the loss of outer hair cells due to atherosclerosis, which led to both low-frequency and high-frequency hearing loss, respectively.^[31]^ Beyond cochlear ischemia, ASCVD may also represent a broader systemic vascular condition that affects the auditory system. The cochlea has high metabolic demands and depends on a delicate microvascular supply; therefore, even subtle endothelial dysfunction, impaired microcirculation, or chronic low-grade inflammation may disturb cochlear homeostasis and auditory signaling.^[32,33]^ These vascular and inflammatory changes may partly explain why hearing loss was associated with prevalent ASCVD in our study. For tinnitus, the mechanism may be more complex. Tinnitus is often accompanied by sleep disturbance, stress, and heightened autonomic arousal, all of which may be linked to adverse cardiovascular profiles.^[34]^ Therefore, hearing loss and tinnitus may not simply be isolated auditory symptoms but may also indicate a subgroup with a higher burden of vascular and systemic health problems. However, given the cross-sectional nature of the present study, these mechanisms remain speculative and should be examined in future longitudinal and mechanistic studies. From a clinical perspective, our findings suggest that individuals with hearing loss, tinnitus, or both conditions may warrant greater attention to cardiovascular health assessment. Future prospective multicenter studies are needed to clarify the temporal relationship among hearing loss, tinnitus, and ASCVD and to determine whether integrated audiological and cardiovascular assessment may benefit selected high-risk populations.

The advantages of this study are as follows: the use of a large, well-characterized NHANES sample, which improves statistical power and sample diversity; the implementation of standardized hearing tests and ASCVD diagnostic criteria; and furthermore, the rigor of our analysis is underscored by the use of multivariate models with sequential adjustments for multiple confounders, complemented by detailed subgroup analyses.

This study presents several limitations. First, the cross-sectional design of this study precludes the ability to establish causal or temporal relationships between hearing loss, tinnitus, and ASCVD. Second, the diagnoses of ASCVD and the presence of hearing loss and tinnitus were primarily based on self-reported questionnaire data. This reliance on self-reporting inevitably introduces potential recall bias and classification bias. Consequently, subclinical ASCVD or mild, unperceived hearing impairment might have been misclassified, which could lead to an underestimation or overestimation of the true strength of the observed associations. Third, NHANES sampling weights were not applied in this study. Consequently, although we adjusted for key demographic variables, our findings may not be fully generalizable to the entire US population. Finally, owing to database constraints, certain unmeasured confounding factors associated with auditory impairment and ASCVD could not be incorporated. Nevertheless, our findings indicate a significant and clinically relevant association between ASCVD, hearing loss, and tinnitus. Future prospective cohort studies utilizing objective clinical measurements and broader international populations are warranted to validate these findings, establish causality, and explore the underlying mechanisms. Future studies should increase the sample size by incorporating additional countries and regions to improve the generalizability and applicability of the findings.

## 5. Conclusion

Hearing loss and tinnitus were independently associated with higher odds of prevalent ASCVD among US adults. Prospective longitudinal studies are needed to clarify the temporal nature of these associations.

## Acknowledgments

The authors appreciate the National Center for Health Statistics at the Centers for Disease Control and Prevention for sharing the data.

## Author contributions

**Methodology:** Xuhui Huang.

**Conceptualization:** Yangyang Chen.

**Software:** Huimin Zhang.

**Validation:** Yao Zeng.

**Data curation:** Xushan Chen.

**Supervision:** Xiaona Tang.

**Writing – original draft:** Luniao Ma.

**Writing – review & editing:** Rucheng Huang.
